# IκBζ Regulates Human Monocyte Pro-Inflammatory Responses Induced by *Streptococcus pneumoniae*

**DOI:** 10.1371/journal.pone.0161931

**Published:** 2016-09-06

**Authors:** Kruthika Sundaram, Mohd. Akhlakur Rahman, Srabani Mitra, Daren L. Knoell, Shireen A. Woodiga, Samantha J. King, Mark D. Wewers

**Affiliations:** 1 Pulmonary, Allergy, Critical Care and Sleep Medicine, Davis Heart and Lung Research Institute, Department of Internal Medicine, Ohio State University Medical Center, Columbus, Ohio, United States of America; 2 Department of Pharmacy, College of Pharmacy, Ohio State University, Columbus, Ohio, United States of America; 3 Center for Microbial Interface Biology, Ohio State University, Columbus, Ohio, United States of America; 4 Center for Microbial Pathogenesis, Research Institute at Nationwide Children's Hospital, Columbus, Ohio, United States of America; 5 Department of Pediatrics, Ohio State University, Columbus, Ohio, United States of America; Louisiana State University, UNITED STATES

## Abstract

Pneumococcal lung infections represent a major cause of death worldwide. Single nucleotide polymorphisms (SNPs) in the *NFKBIZ* gene, encoding the transcription factor IκBζ, are associated with increased susceptibility to invasive pneumococcal disease. We hence analyzed how IκBζ might regulate inflammatory responses to pneumococcal infection. We first demonstrate that IκBζ is expressed in human blood monocytes but not in bronchial epithelial cells, in response to wild type pneumococcal strain D39. D39 transiently induced IκBζ in a dose dependent manner, with subsequent induction of downstream molecules involved in host defense. Of these molecules, IκBζ knockdown reduced the expression of IL-6 and GMCSF. Furthermore, IκBζ overexpression increased the activity of IL-6 and GMCSF promoters, supporting the knockdown findings. Pneumococci lacking either pneumolysin or capsule still induced IκBζ. While inhibition of TLR1/TLR2 blocked D39 induced IκBζ expression, TLR4 inhibition did not. Blockade of p38 MAP kinase and NFκB suppressed D39 induced IκBζ. Overall, our data demonstrates that IκBζ regulates monocyte inflammatory responses to *Streptococcus pneumoniae* by promoting the production of IL-6 and GMCSF.

## Introduction

Pneumonia is one of the leading causes of death around the world, especially in children [1, 2]. Among the various agents that cause pneumonia, *Streptococcus pneumoniae* is the commonest [2, 3]. It is a Gram positive, facultative anaerobic bacterium that is pathogenic. It predominantly colonizes in the upper airway tract asymptomatically but it can also spread to other sites including the brain, blood and the middle ear to cause disease [4]. Many components of the bacterium act as virulence factors, contributing to its pathogenicity, including its polysaccharide capsule, the pore forming toxin pneumolysin, the autolytic enzyme LytA and the choline binding proteins anchored to the cell wall [5–7]. Although airway epithelial cells act as the primary site of pneumococcal colonization, innate immune cells in the lungs such as monocytes and macrophages can sense the bacteria and mount an immune response to protect the host. In this context, monocyte influx into an infected lung is well documented [8–10]. At the molecular level, the pathogen is sensed by various pathogen recognition receptors (PRRs) including Toll-like receptors (TLRs) and NOD-like receptors (NLRs) expressed by the phagocytes [11, 12]. The signaling of these PRRs culminate in the activation of nuclear factor κB (NFκB) and the release of inflammatory cytokines [10, 13, 14] such as TNF, IL-1β, and IL-6, resulting in an early innate immune response that is required for infection control and for host defense [12, 15]. It is hence important to understand the molecular mechanisms underlying the host-pathogen interaction to improve strategies of effectively tackling pneumococcal pneumonia.

*NFKBIZ* is a primary response gene that is induced rapidly in monocytes and macrophages in response to LPS [16–18]. It encodes the protein IκBζ, also called MAIL or INAP [16–19], which is a transcription factor that binds to NFκB, leading to regulation of several secondary response genes such as *IL6*, *IL12*, *LCN2*, *IFNG* and *DEFB4A* [17, 20–24]. The molecule belongs to the IκB family since it contains multiple ankyrin repeat sequences at its carboxy-terminus with which it binds to NFκB subunits [16, 18]. The amino-terminus of the protein contains a transcriptional activation domain and a nuclear localization sequence. Unlike IκBα that is constitutively expressed in the cytoplasm to keep NFκB subunits sequestered from nuclear translocation [19], IκBζ is an inducible protein that binds to NFκB inside the nucleus. IκBζ binds to the promoter sequences of secondary response genes by forming a complex with either p50 homodimers or p50-p65 heterodimers of NFκB to cause transcriptional regulation. Although IκBζ has a demonstrated inhibitory role [18], its function as a transcriptional activator dominates. Apart from monocytes, IκBζ is also induced in epithelial cells in response to cytokines such as IL1β, IL-18 and IL-17 [21, 22, 25, 26]. There are two known isoforms of IκBζ: IκBζ-long (IκBζ-L) and IκBζ-short (IκBζ-S), of which IκBζ-L protein is predominantly expressed [27].

IκBζ has been studied as a key regulator of innate immune responses associated with several lung inflammatory disorders [25, 28]. Importantly, polymorphisms in the *NFKBIZ* gene have been linked to increased susceptibility to invasive pneumococcal disease [29]. We therefore chose to investigate how IκBζ may regulate inflammatory responses to pneumococcal infection. This is especially important since IκBζ knockout mice display impaired mucosal function in their skin and eyes [30, 31], indicating a key defense function for IκBζ at barrier sites. We have demonstrated that in human monocytes, IκBζ regulates the expression of IL-6 and GMCSF in response to D39, a wild type strain of *S*.*pneumoniae*. We further determined the mechanism underlying the IκBζ mediated response, by identifying factors of the pathogen and of the host responsible for IκBζ expression and function.

## Materials and Methods

### Reagents and antibodies

The reagents were obtained from the following sources: purified LPS from *Escherichia coli* strain 0111:B4 (Enzo Life Sciences), PamCSK4 from EMC microcollections, CD14 beads (MiltenyiBiotec), RPMI1640 (Cellgro), fetal bovine serum (FBS) (Atlanta Biologicals), penicillin-streptomycin (Invitrogen), and endotoxin free bovine serum albumin (BSA) (MP Biomedicals), Bronchial Epithelial Growth Medium BEGM bullet kit (Lonza), Bronchial Air Liquid Interface B-ALI media (Lonza), bovine collagen type I (Corning), fibronectin (Corning), LPS from *Rhodobacter sphaeroides* (RS-LPS) (Invivogen), CUCPT22 (Tocris Bioscience), SB203580 (Sigma-Aldrich) and JSH23 (Calbiochem). Scrambled siRNA control and siIκBζ (sequence UGAUGGACCUGCUUGCAAA) were purchased from Dharmacon Thermo Scientific. Rabbit antiserum against IκBζ was generated in our laboratory using recombinant protein expressed in *E*. *coli* [17]. Beta-actin antibody (monoclonal clone C4) and HSP 90α/β antibody (mouse monoclonal) were purchased from MP Biomedicals and Santa Cruz Biotechnology respectively.

### Bacterial culture and multiplicity of infection (MOI) calculation

All the strains of *S*. *pneumonia*e used: wild type D39, mutant lacking capsule (D39Δcap) and mutant lacking pneumolysin (D39Δ*ply*) were generated in Dr. Samantha King’s laboratory. The strains were cultured on trypticase soy agar plates with 5% sheep blood (Thermo Scientific) overnight at 37°C with 5% CO_2_. Bacteria were harvested in sterile PBS, washed and enumerated by serial-dilution. The optical density of the culture at 600nm was then correlated to the number of live bacterial colony forming units (cfu). An OD_600_ of 0.04 was thus equivalent to 25x10^4^cfu/ml, for all the strains used. The OD_600_ values and their CFUs correlated consistently across all experiments. An MOI of 0.1 means 1 bacterium per 10 monocytes.

### Mammalian cell culture

Primary human bronchial epithelial cells (HBECs), purchased from Lonza were allowed to differentiate in B-ALI media in 24-well inserts (6.5mm) as per manufacturer’s instructions. These cells (5x10^4^ cells/33mm^2^) were treated with D39 at an MOI of 0.1 or 1, or rhIL-1β (10ng/ml). Monocytes were obtained from healthy subjects after informed consent following a protocol approved by the Ohio State University Institutional Review Board or from fresh buffy coats purchased from the American Red Cross. Monocytes were purified from blood by Histopaque density gradient centrifugation using lymphocyte separation media (Cellgro) followed by CD14 positive selection as described previously [17]. Purified monocytes were maintained in RPMI1640 supplemented with 10% FBS in a 37°C humidified incubator with 5% CO_2_. Two million monocytes at 10^6^ cells/ml concentration were used per condition in all experiments, unless specified otherwise. The cells were treated with inhibitors at the specified concentrations, 30 minutes before infection with pneumococci. LPS (10ng/ml) or PamCSK4 (5ng/ml) were used as positive controls. The cells were then washed 2–3 times in PBS and lysed to obtain total protein for immunoblotting or to obtain RNA for quantitative PCR. Cell supernatants were analyzed by ELISA for cytokine release.

### Luciferase reporter assay

For IL-6 promoter activation studies, HEK 293 cells were transfected with pGL3 basic (Promega), pIL-6 Luc (generous gift from Dr. Oliver Eickelberg, Germany) along with pCDNA3.1+ or IκBζ-L pCDNA 3.1+ using Lipofectamine 2000 (Invitrogen). For GMCSF promoter activation, the cells were transfected with pXPG or pGMCSF (generous gift from Dr. Peter Cockerill, UK) along with IκBζ plasmids. pEGFPc2 and Renilla luciferase plasmid pRLTK were used as the transfection controls for IL-6 and GMCSF respectively. The cells were harvested and then lysed in passive lysis buffer (Promega) 36h after transfection. The dual luciferase reporter assay system (Promega) was used to analyze the cell lysates for firefly luciferase and renilla luciferase activity. For IL-6, luciferase expression was normalized to the expression of transfection control EGFP from protein-normalized immunoblots.

### Small interfering RNA (siRNA) mediated knockdown of IκBζ

Monocytes (5x10^6^ cells/well) were nucleofected with 100pmol scrambled siRNA control or siRNA specific to IκBζ, using the Amaxa kit (Lonza) designed for human monocytes. The cells were then plated on 6 well plates and allowed to rest overnight. They were then infected with D39 (MOI 0.1) for 3 and 6 h.

### Preparation of cell lysates and immunoblotting

Cells were lysed in TN1 lysis buffer (50mM Tris-HCl, pH 8.0, 125mM NaCl, 10mM ethylene-diamine-tetra-acetic-acid (EDTA), 10mM NaF, 10mM sodium pyrophosphate, 1% Triton X-100) with a protease inhibitor cocktail mix (Sigma-Aldrich), 10mM methoxysuccinyl-Ala-Ala-Pro-Val-chloromethyl ketone, 1mM phenylmethylsulfonyl fluoride (Sigma-Aldrich), and 3mM sodium orthovanadate. The extracts were incubated on ice for 15 minutes, syringed 10–15 times and centrifuged at 16,000g for 10 minutes. The lysates were transferred to a new Eppendorf tube and total protein in each sample was determined using Lowry assay (Bio-Rad). Equal protein (20μg per lane) was loaded onto a NuPAGE 7% Tris-acetate gel (Invitrogen). The separated proteins were transferred onto polyvinylidene difluoride membranes. The membranes were blocked with 10% nonfat milk, incubated overnight at 4°C with the primary antibody, washed and then stained with appropriate peroxidase conjugated secondary antibody for an hour. The protein bands were visualized by enhanced chemiluminescence substrate (GE Healthcare) by autoradiography. The expression of IκBζ protein was quantified by normalizing its densitometry readings with those of house-keeping proteins HSP90 or β-actin. For all the immunoblots, the primary antibody dilutions used for anti-IκBζ antiserum, HSP90 antibody and β-actin antibody were 1:2000, 1:2000 and 1:10,000 respectively.

### ELISA

ELISA kit for IL-6 and IL-8 was purchased from eBioscience, for GMCSF from BD Biosciences.

### Quantitative PCR

The cells were treated with Trizol and total RNA was extracted using the manufacturer’s protocol (Invitrogen). cDNA made from the total RNA (~1 to 2μg) using Thermoscript reverse transcriptase (Invitrogen) was used as the template for quantitative polymerase chain reaction (qPCR) by the SYBR Green method as previously described [32]. Briefly, relative copy number (RCN) of the genes was calculated using the equation: RCN = E –^ΔCt^ × 100, where E is the efficiency of PCR and ΔCt = Ct_(target)_ –Ct_(average of two housekeeping genes)_. GAPDH and CAP-1 (cAMP-accessory protein) were the two house-keeping genes used. Efficiency of PCR was calculated using the equation: E = 10^(–1/slope)^ and based on our preliminary experiments, the value of E was observed to be nearly 2, which represents almost 100% efficiency for all selected genes in PCR, including housekeeping genes. Hence, we substituted 2 for E while calculating RCN. Primers specific to the different genes (sequences in **S1 Table**) were used to evaluate mRNA expression. Gene expression was normalized to the average of two house-keeping genes GAPDH and CAP-1. Values are expressed as relative copy numbers RCN.

### Statistical analysis

All experiments were repeated at least three times for reproducibility of results. Results are expressed as mean ± SEM. All experiments involved two group comparisons and hence were analyzed using Student’s paired, two-tailed t-test with block design (SAS-JMP) as in our previous study [28]. The block design takes into account donor variability across experiments while calculating the p-value. Significance was defined as *p* < 0.05.

## Results

### Monocytes but not bronchial epithelial cells express IκBζ in response to *S*.*pneumoniae*

**Fig 1:**
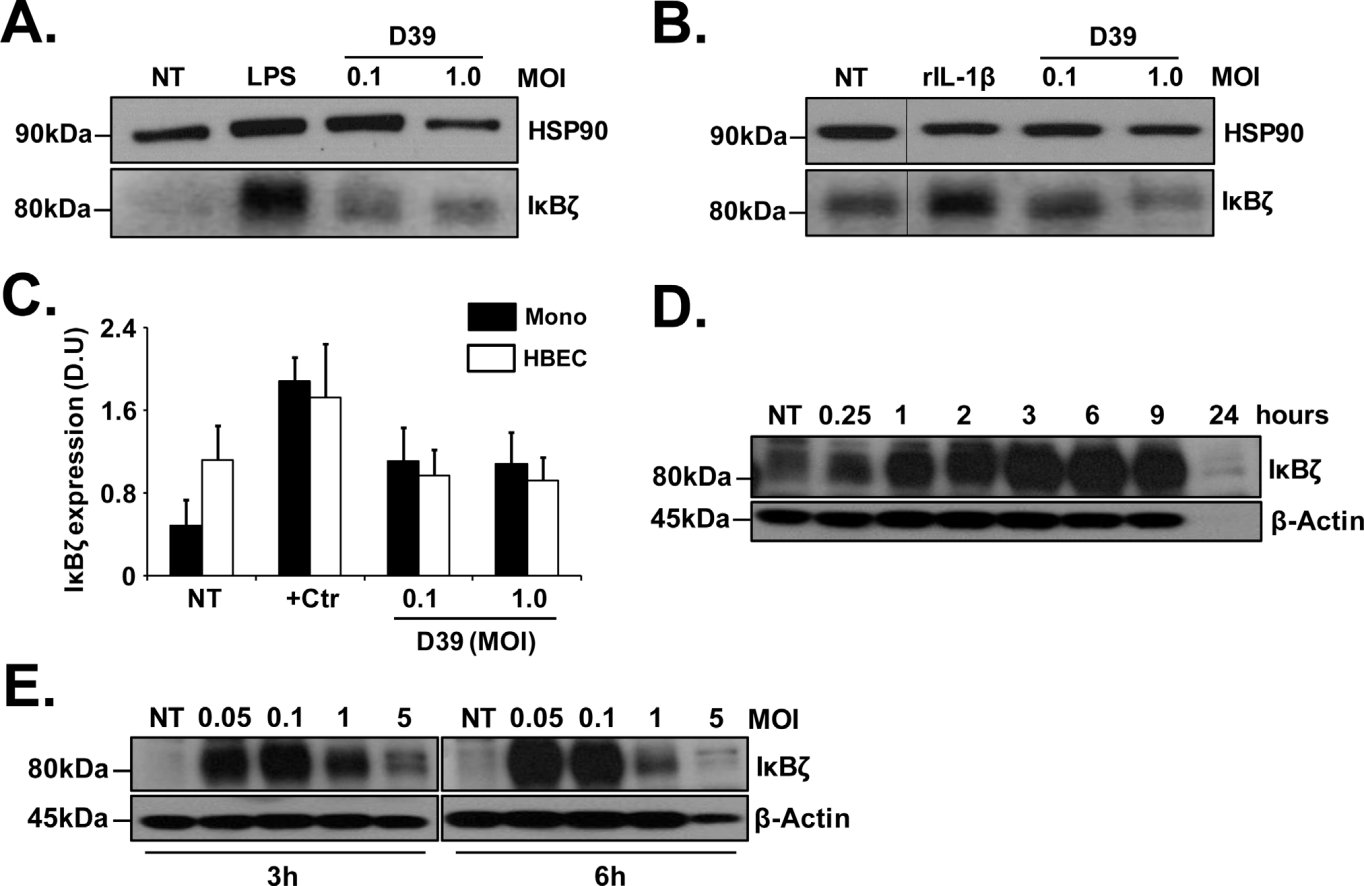
Pneumococcus induced IκBζ expression in monocytes. **(A)** Human monocytes (10^6^ cells/ml) or **(B)** HBECs (5X10^4^ cells/33 mm^2^) were infected with pneumococcus strain D39 at MOIs 0.1 and 1.0 for 3 h. LPS (1μg/ml) and rIL-1β (10ng/ml) were used as positive controls (+Ctr) for monocytes and HBECs respectively. **(C)** Densitometry graph for IκBζ expression normalized to house-keeping proteins HSP90 or β-actin in monocytes from blot (A) and in HBECs from blot (B). **(D)** Human monocytes (10^6^ cells/ml) were infected with D39 at MOI 0.1 for different time points through 24 h or **(E)** with different MOIs of D39 for 3 and 6h. Cell extracts were analyzed using immunoblotting with anti-IκBζ antiserum, HSP90 antibody and β-actin antibody. The immunoblots represent 3 independent experiments. NT stands for not treated. Vertical lines have been inserted to indicate a repositioned gel lane.

Adherence of *S*. *pneumoniae* to airway epithelial cells has been demonstrated to be a critical step in the colonization and infection of the host [33–35] while mononuclear phagocytes are rapidly recruited into infected lungs [8–10]. Hence, we evaluated if IκBζ expression is induced in human bronchial epithelial cells and in blood monocytes in response to *S*. *pneumoniae*. Primary HBECs or monocytes purified from peripheral blood were infected with pneumococcal strain D39, at MOIs 0.1 and 1 for 3 h. D39 induced IκBζ expression in monocytes (**Fig 1A and 1C**), similar to the positive control LPS. Interestingly, HBECs had background IκBζ expression that was further induced only by the positive control rIL-1β but not by D39 (**Fig 1B and 1C**). Thus, in response to D39, IκBζ expression is induced in monocytes but not bronchial epithelial cells.

### IκBζ expression is induced in monocytes in response to pneumococcus in a dose dependent manner

To determine the expression pattern of IκBζ in monocytes following pneumococcal infection, monocytes were treated either with D39 (MOI 0.1) for different time points for a period of 24 h or with different MOIs of D39 for 3 and 6 h. Cells were lysed to determine mRNA expression using real-time qPCR and protein expression using immunoblotting. IκBζ mRNA levels increased with increasing time (**S1A Fig**) or with increasing dose of D39 (**S1B Fig**) but were almost undetectable in the non-treated controls. IκBζ protein expression peaked between 3 and 6 h after infection (**Fig 1D**) at an MOI of 0.1. The expression of IκBζ protein was more pronounced with MOI 0.1 at 3h post-infection (**Fig 1E**), although both MOIs 0.05 and 0.1 caused induction. However, longer time points, e.g. 24h (**Fig 1D**) and higher MOIs (**Fig 1E**) reproducibly diminished the IκBζ, presumably from the toxic effects of D39 overgrowth.

### IκBζ knockdown leads to decreased expression of IL-6 and GMCSF

**Fig 2:**
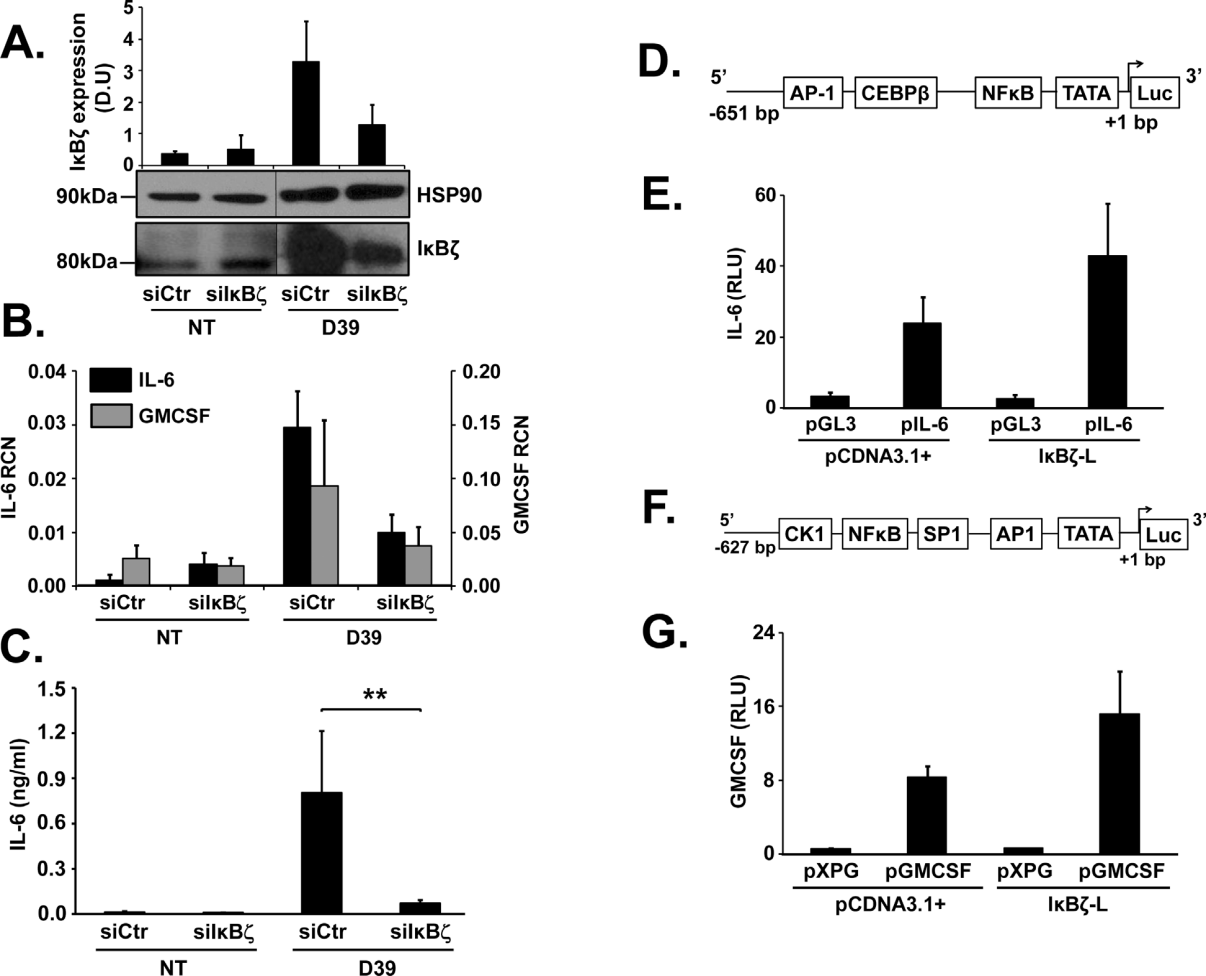
IκBζ regulates D39 induced IL-6 and GMCSF. Human monocytes (10^6^ cells/ml) were nucleofected with scrambled siRNA control (siCtr) or siIκBζ followed by infection with D39 at an MOI of 0.1 for 3 and 6 h. **(A)** Cell extracts were analyzed using immunoblotting for IκBζ and HSP90 expression and the reduction in IκBζ protein expression was quantified through densitometry readings, where IκBζ expression was normalized to either HSP90 or β-actin. **(B)** qPCR analysis on cell extracts for mRNA expression of IL-6 and GMCSF. **(C)** IL-6 release from the cells was analyzed using ELISA. Maps of promoter luciferase reporter constructs are represented for **(D)** IL-6 and **(F)** GMCSF. HEK293 cells (2X10^5^ cells/ml) were transfected with pcDNA3.1+ or IκBζ along with **(E)** pGL3 or pIL-6 with pEGFPc2 as transfection control or **(G)** pXPG or pGMCSF with pRLTK as transfection control. After 48 h, the cells were lysed and the extracts were subjected to luciferase assay. The immunoblot represents 3 independent experiments. The bar graphs represent the mean ± SEM of 3–4 and 2–3 independent experiments for luciferase, ELISA assays and qPCR respectively. ** p = 0.03. NT stands for not treated. Vertical lines have been inserted to indicate repositioned gel lanes.

To elucidate the function of IκBζ as a transcriptional regulator of pneumococcus induced downstream factors involved in host defense, monocytes were nucleofected with siRNA specific to IκBζ (siIκBζ) and subsequently infected with D39 at an MOI of 0.1 for 3 and 6 h. siRNA mediated knockdown of IκBζ resulted in ~50% reduction in its expression following treatment with D39 (**Fig 2A**) as well as the positive control PamCSK4 (TLR1/2 agonist) (**S2A Fig**), as confirmed using immunoblotting and quantification of expression using densitometry (**Fig 2A**). Of the downstream genes that were tested (**S2C Fig**), we observed suppression in the mRNA expression of pneumococcus induced GMCSF and IL-6 (**Fig 2B**) and the release of IL-6 protein (**Fig 2C**), in the presence of siIκBζ as compared to scrambled siRNA control. This response was specific since siIκBζ did not affect D39-induced IL-8 expression (**S2B Fig**), as observed in previous studies [24, 36]. Therefore, IκBζ is a modulator of monocytic IL-6 and GMCSF, in response to pneumococcal challenge.

### IκBζ increases promoter activity of IL-6 and GMCSF

To demonstrate direct regulatory effects of IκBζ on the gene expression of IL-6 and GMCSF, we employed a luciferase reporter assay with IL-6 or GMCSF promoter constructs (**Fig 2D and 2F**). We co-transfected HEK293 cells with IL-6 or GMCSF promoter luciferase constructs and IκBζ pcDNA3.1+. The promoter constructs in combination with the control vector pcDNA3.1+ had basal luciferase expression. However, with IκBζ overexpression, there was almost a 2 fold increase in the luciferase expression (**Fig 2E and 2G**). Thus, IκBζ enhances promoter activity of IL-6 and GMCSF, further supporting its role as a transcriptional regulator of the *S*.*pneumoniae* induced immune response.

### Cytokine kinetics following pneumococcal infection

**Fig 3:**
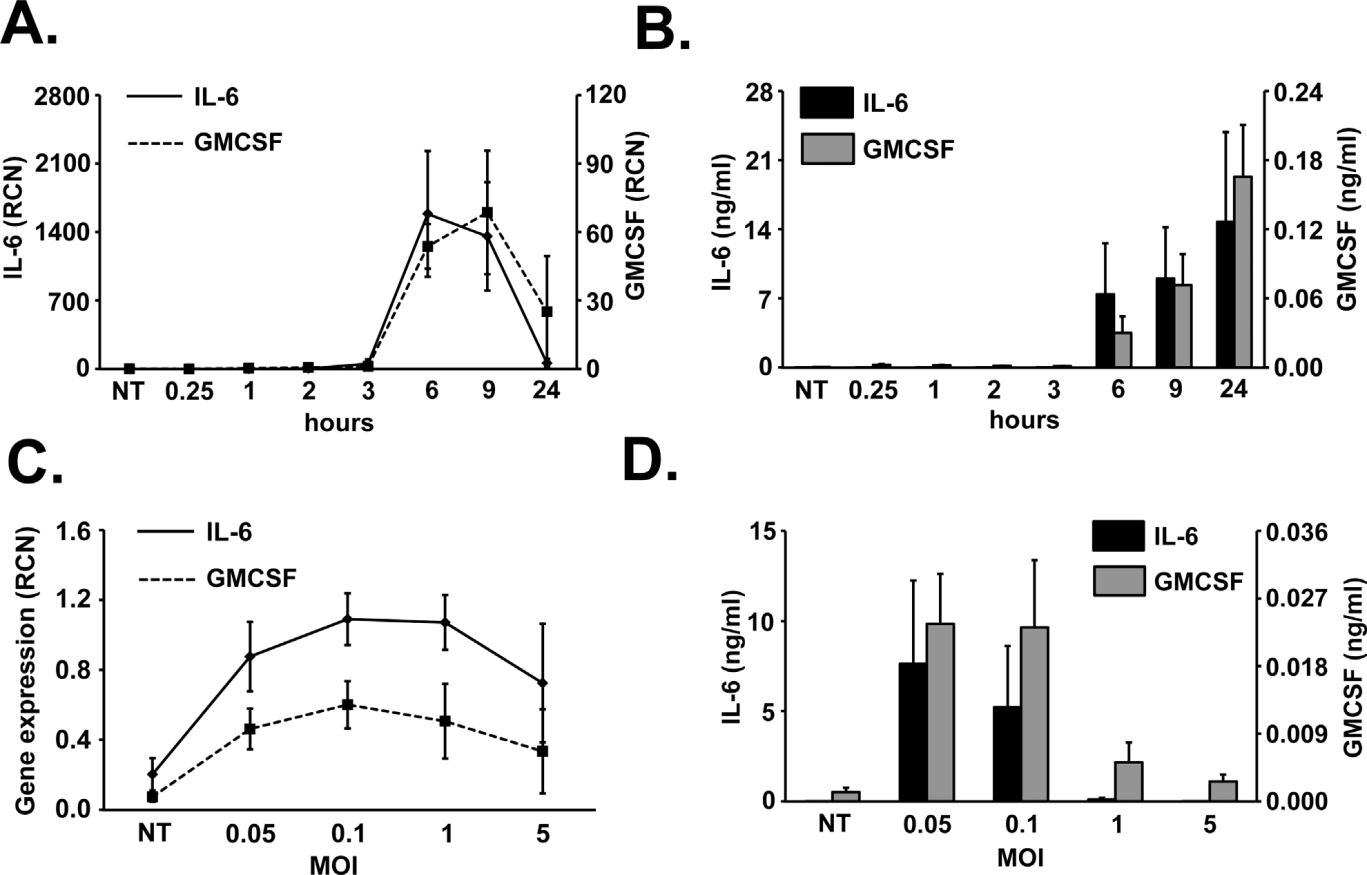
Cytokine kinetics in monocytes in response to D39. Human monocytes (10^6^ cells/ml) **(A, B)** were infected with D39 at an MOI of 0.1 for different time periods through 24 h or were infected with different MOIs of D39 for **(C)** 3 h or **(D)** 6 h. Cell extracts were analyzed for **(A, C)** mRNA expression using qPCR or **(B, D)** cytokine release using ELISA. The graphs represent the mean ± SEM of 3 independent experiments. NT stands for not treated.

We then examined the mRNA and protein expression of D39 induced IL-6 and GMCSF, regulated by IκBζ as shown in the previous sections. The gene expression of these cytokines was induced by 3 h and peaked at 6 to 9 h post pneumococcal infection (**Fig 3A**). We then evaluated the effect of different MOIs of D39 on the expression of IL-6 and GMCSF and found that an MOI of 0.1 caused maximum induction of these genes (**Fig 3C**). The release of the cytokines was detectable starting at 6 h of infection (**Fig 3B**) and in response to MOIs 0.05 and 0.1 (**Fig 3D**). The delayed cytokine response following the early IκBζ response further corroborates our hypothesis that IκBζ is an early response gene regulator of *S*. *pneumoniae* induced IL-6 and GMCSF. Based on these observations, we hence used 0.1 MOI of D39 as the optimum dose, and 3 h of infection as the optimal time to observe IκBζ protein expression and downstream gene expression, a measure for the transcriptional activity of IκBζ, in all the following experiments, unless specified otherwise. Additionally, we also measured IL-6 protein release at 6 h post infection, the earliest time point to observe detectable amount of the cytokine.

### Capsule and pneumolysin are dispensable pathogenic factors of *S*. *pneumoniae* for IκBζ induction

**Fig 4:**
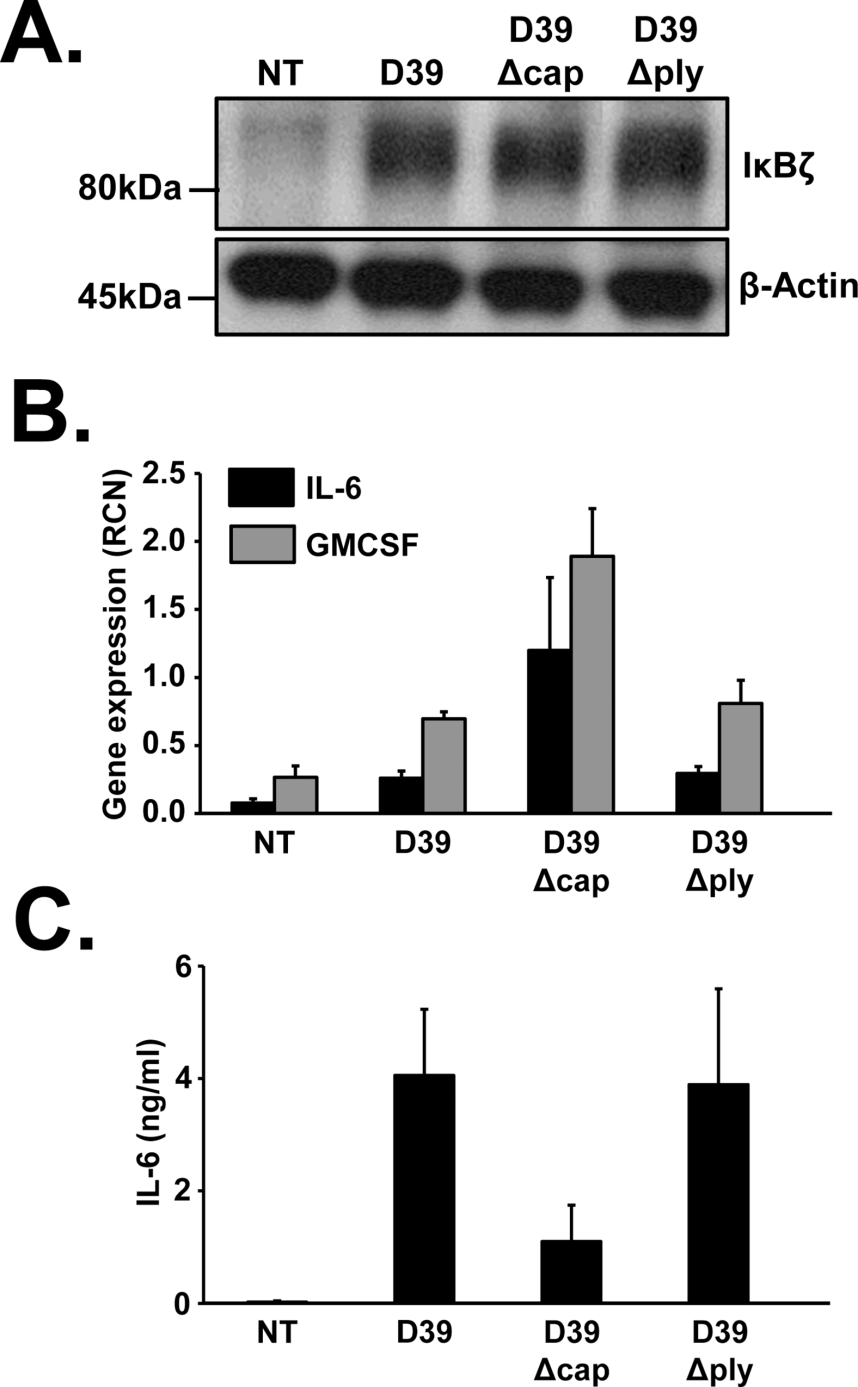
Capsule and pneumolysin of D39 are dispensable for IκBζ induction. Human monocytes (10^6^ cells/ml) were infected with D39, D39Δcap or D39Δply at an MOI of 0.1 for 3 and 6 h. Cell extracts were analyzed at 3h **(A)** using immunoblotting for IκBζ and actin expression **(B)** using qPCR for mRNA expression. **(C)** IL-6 release from the cells was analyzed using ELISA after 6h of infection. The immunoblot represents 3 independent experiments and the bar graphs represent the mean ± SEM of 3 independent experiments. NT stands for not treated.

Having demonstrated the function of IκBζ in regulating pneumococcal induced IL-6 and GMCSF expression, we were curious to explore the mechanism underlying the induction of IκBζ expression. Various cellular components of *S*. *pneumoniae* have been demonstrated to be potent pathogenic factors, modifying inflammatory responses. They include the toxin pneumolysin and the bacterial capsule [5]. We therefore evaluated the effect of these factors on the expression of IκBζ and downstream cytokines. Monocytes were infected with wild type D39, D39Δcap (lacking the capsule), and D39Δ*ply* (lacking pneumolysin), each at an MOI of 0.1 for 3 and 6 h. Like the wild type strain D39, the mutants also induced protein expression of IκBζ (**Fig 4A**), mRNA levels of IL-6 and GMCSF (**Fig 4B**) and IL-6 release (**Fig 4C**). Although the induction by each strain varied somewhat, these results demonstrate that pneumococcal capsule and pneumolysin are dispensable for IκBζ mediated transcriptional responses in monocytes.

### D39 induced expression of IκBζ, IL-6 and GMCSF is inhibited following TLR1/TLR2 but not TLR4 blockade

**Fig 5:**
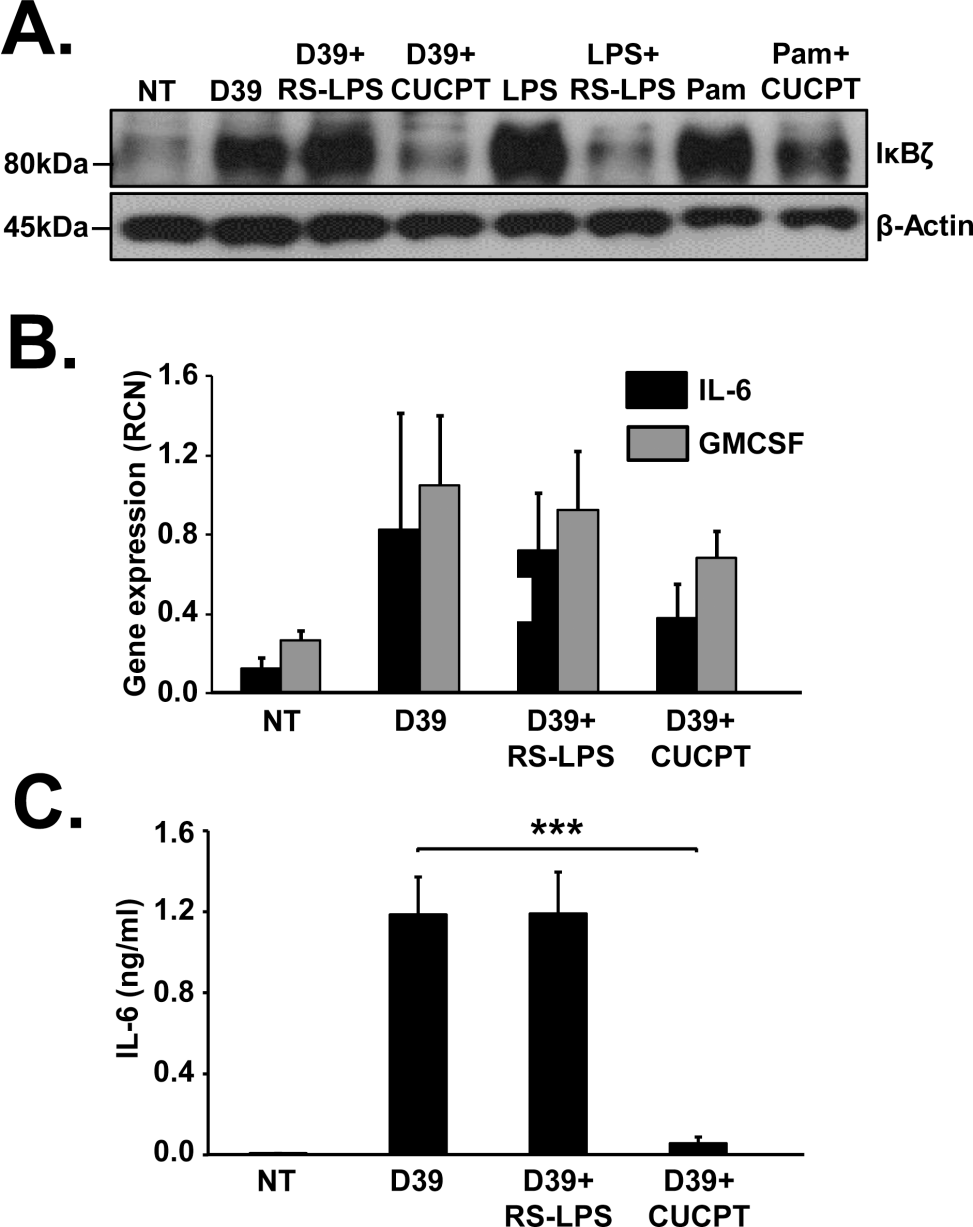
TLR1/TLR2 but not TLR4 blockade inhibits IκBζ induction. Human monocytes (10^6^ cells/ml) were pretreated with RS-LPS (10μg/ml) or CUCPT22 (50uM) for 30 minutes followed by infection with D39 at an MOI of 0.1 for 3 and 6 h. LPS (10ng/ml) and PamCSK4 (Pam) (5ng/ml) were used as positive controls for TLR4 and TLR1/TLR2 induced IκBζ respectively. Cell extracts were analyzed **(A)** using immunoblotting for IκBζ and actin expression **(B)** using qPCR for mRNA expression. **(C)** IL-6 release from the cells was analyzed using ELISA. The immunoblot represents 4–5 independent experiments and the bar graphs represent the mean ± SEM of 4–5 independent experiments. ***p<0.01. NT stands for not treated.

The two most common receptors known to sense pneumococcal pathogenic factors are TLR2 and TLR4 [12]. To identify the surface receptor activated by D39 to induce IκBζ expression, we treated monocytes with a TLR1/TLR2 inhibitor, CUCPT22 or a TLR4 antagonist, LPS obtained from *Rhodobacter spheroides* (RS-LPS). CUCPT22 inhibited the expression of IκBζ induced by D39, while RS-LPS did not (**Fig 5A**). Additionally, TLR1/TLR2 blockade led to a decrease in the gene expression of IL-6 and GMCSF (**Fig 5B**) and in the release of IL-6 protein (**Fig 5C**). These findings suggest that the IκBζ mediated immune response is due to D39 sensing by TLR1/TLR2 dimer and not TLR4.

### p38 MAP kinase and NFκB are upstream regulators of pneumococcus induced IκBζ

**Fig 6:**
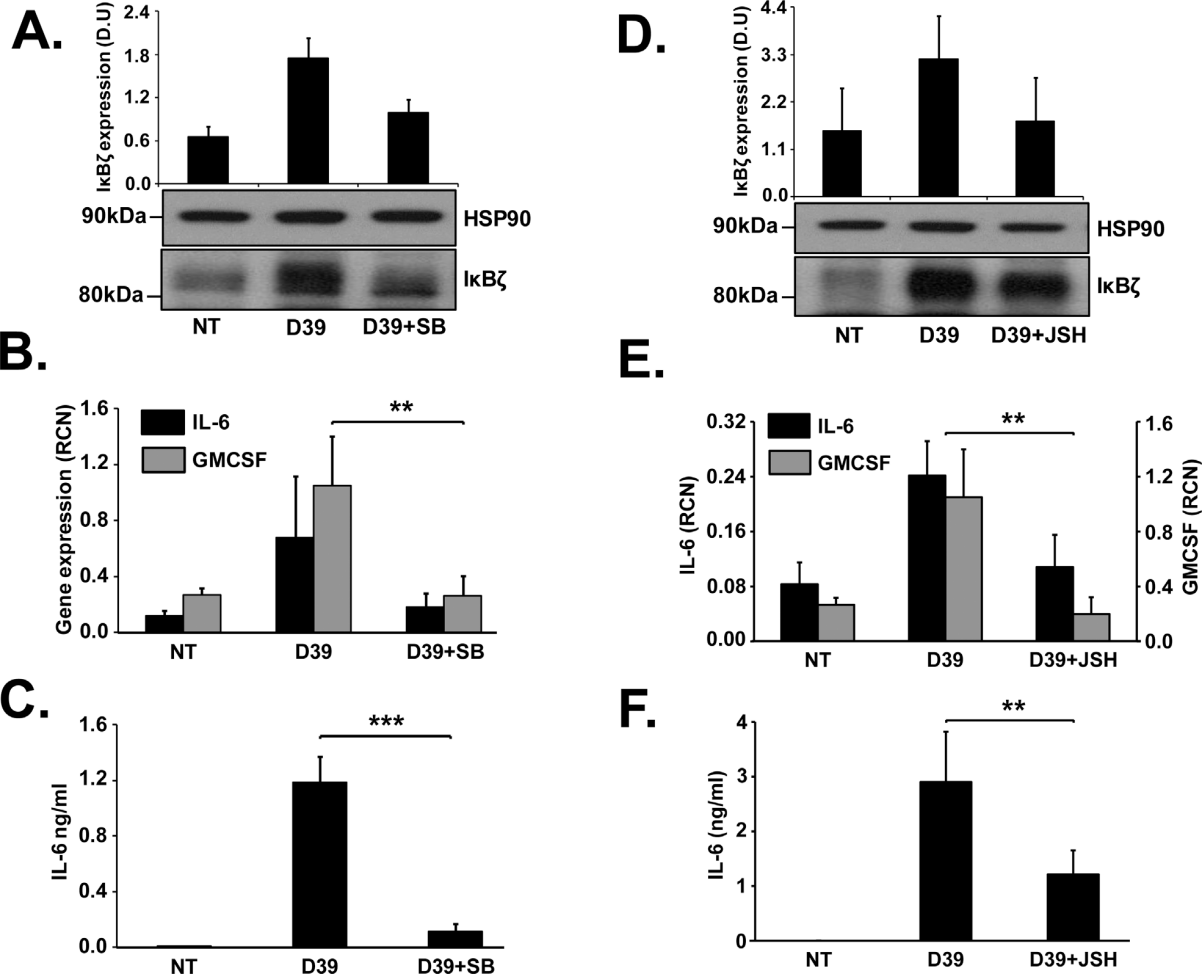
Upstream regulators of IκBζ mediated immune response to pneumococcus. Human monocytes (10^6^ cells/ml) were **(A-C)** pretreated with the p38 MAP kinase inhibitor, SB203580 (SB; 10uM) or **(D-F)** the NFKB inhibitorJSH23 (JSH; 40uM) for 30 minutes followed by infection with D39 at an MOI of 0.1 for 3 and 6 h. **(A,D)** Cell extracts were analyzed using quantification of protein expression by immunoblotting for IκBζ and loading control HSP90. **(B,E)** qPCR analysis for mRNA expression of IL-6 and GMCSF. **(C,F)** IL-6 release from the cells was analyzed using ELISA. The immunoblot represents 4–5 independent experiments and the bar graphs represent the mean ± SEM of 4–5 independent experiments. ** p≤ 0.03, *** p<0.01. NT stands for not treated.

TLR2 signaling has been demonstrated to cause the activation of p38 MAP kinase (p38 MAPK) [37] that then regulates inflammatory responses by phosphorylating transcription factors like NFκB and AP-1 [38, 39] that are known binding partners of IκBζ [36]. We treated monocytes with the p38 MAPK inhibitor SB203580 or the NFκB inhibitor JSH23 for 30 minutes, followed by infection with D39. The inhibitors blocked the expression of IκBζ protein (**Fig 6A and 6D**), the mRNA expression of IL-6 and GMCSF (**Fig 6B and 6E**) and the release of IL-6 (**Fig 6C and 6F**). Thus p38 MAP kinase and NFκB are upstream activators of the IκBζ mediated inflammatory response to pneumococcal infection.

## Discussion

IκBζ is a known partner of NFκB that regulates several factors involved in host defense [21, 22] and is implicated as a critical player in the pathogenesis of several disorders [25, 28, 30, 40]. Interestingly, multiple single nucleotide polymorphisms on the *NFKBIZ* gene are associated with increased susceptibility to invasive pneumococcal disease [29]. But, IκBζ expression and function in response to *S*. *pneumoniae* infection remain unexplored. We demonstrate for the first time, a potential role for IκBζ in mediating inflammatory responses to *S*.*pneumoniae* (**S3 Fig**). We show that in response to the pneumococcal strain D39, IκBζ expression is induced in human blood monocytes but not in airway epithelial cells. This was unexpected and interesting since airway epithelial cells are one of the main sites of pneumococcal interaction with the host during mucosal infection [33, 41, 42]. However, these epithelial cells have been shown to lack the expression of TLRs [43], receptors that trigger the IκBζ mediated cytokine expression in response to *S*.*pneumoniae*. The reason behind the baseline expression of IκBζ observed in bronchial epithelial cells remains unexplained.

Typical of its expression in immune cells, IκBζ protein was induced in monocytes in response to the wild type pneumococcus strain D39 [24, 36, 44]. However, mRNA expression of IκBζ was sustained through the 24 h post infection, indicating possible post-transcriptional regulation. We screened for pneumonia relevant, host defense factors [10, 45–48] that are regulated by IκBζ using RT-PCR (**S2 Fig**). Although there was a subtle decrease in the mRNA levels of IL-1β and lungkine CXCL-5, the expression of IL-6 and GMCSF was significantly reduced with IκBζ knockdown. GMCSF is known to improve lung immunity by accelerating bacterial clearance and neutrophil recruitment [46, 49] while IL-6 contributes to lung defense by orchestrating the pro- and anti-inflammatory cytokine network [50], thus emphasizing the relevance of this study to the pathogenesis of pneumococcal pneumonia.

*NFKBIZ* is a primary response gene that is known to play a vital role in orchestrating crucial early innate responses to immune challenges. In this context, single nucleotide polymorphisms in *NFKBIZ* have been linked with a predisposition to invasive pneumococcal sepsis. Although the mechanism responsible for this association remains unknown, we believe that our findings begin to unravel some of the mechanistic details. Interestingly, out of the two known isoforms of IκBζ, we observed only IκBζ-L protein expression in monocytes. In this context, the *NFKBIZ* SNPs linked to an increased susceptibility to invasive pneumococcal disease, exist in a critical intron containing the splice site responsible for determining whether *NFKBIZ* transcribes the long or short form of IκBζ, i.e., IκBζ-L vs. IκBζ-S [29]. It could hence be speculated that expression of the functionally active IκBζ-L protein is hindered in subjects with the SNPs, thus making them more susceptible to pneumococcal infections. Future studies to address the relative ability to produce IκBζ long and short forms in the context of these SNP variations are planned.

In addition to the physiologically common wildtype strain of *S*.*pneumoniae*, the less common, capsule deficient strains, known to cause conjunctivitis in humans [51], also induce IκBζ mediated inflammatory responses in monocytes. This finding further stresses the importance of IκBζ in pneumococcus induced immune responses.

The mRNA expression of IL-6 and GMCSF followed that of IκBζ, i.e., appropriate kinetics for IκBζ to transcriptionally regulate these cytokines. We used 6 h of infection as the time to observe detectable IL-6 protein amounts to avoid cell death due to pneumococcal overgrowth which was observed at longer time points (MOI: 0.1; **S4 Fig**). We could not detect GMCSF protein release. This is in accordance with our previous demonstration that monocytes produced much less GMCSF than IL-6 [28].

We showed that pneumococcal pathogenic factors, pneumolysin and the capsule, are dispensable for IκBζ induction. The mRNA levels of IL-6 and GMCSF induced by the capsule mutant did not correlate with the expression pattern of IκBζ protein. The reason behind this observation could be the participation of other signaling molecules activated by D39Δcap that contribute to the expression of the cytokines. Nevertheless, the expression of IκBζ, IL-6 and GMCSF was induced even when D39 lacked its capsule or pneumolysin. Based on our findings in Fig 5, it is likely that pneumococcal pathogenic factors known to act through the TLR1/TLR2 complex, such as lipotechoic acid (LTA) [52] are responsible for IκBζ induction.

We demonstrated that pneumococcus is detected by the TLR1/TLR2 receptor complex. CUCPT22, the TLR1/TLR2 inhibitor had non-specific effects on TLR4, since it also blocked LPS induced IκBζ expression (data not shown). However, since RS-LPS, a specific inhibitor of TLR4 [28] had no impact on D39 induced IκBζ expression, we concluded that the IκBζ induction is through TLR1/TLR2 and not TLR4. In accordance with this observation, pneumolysin, that is known to trigger TLR4 signaling [53] was demonstrated to be dispensable for inducing the IκBζ mediated immune response. Although further studies are clearly required to confirm the inhibitor studies, our findings agree with prior reports that link TLR1/TLR2 signaling to pneumococcal host responses [52, 54].

NFκB and p38 MAP kinase were identified as upstream regulators of the IκBζ mediated immune response to pneumococcal infection. Inhibition of these factors resulted in reduced expression of IκBζ mRNA (data not shown) as well as protein, indicating that the regulation is transcriptional. This observation was particularly interesting since NFκB has been demonstrated to be required for LPS induced IκBζ expression, while blocking the various MAP kinases had no effect, indicating that the MAP kinases are dispensable for LPS induced IκBζ expression [55]. Based on our data, it is conceivable that activation of TLR1/TLR2 complex by pneumococcus results in activation of both signaling branches of the MyD88-TRAF6-TAK1 pathway, namely the MAP kinases and subsequently the transcription factor AP-1, and the IKKs and subsequently NFκB, especially since AP-1 and NFκB are known to be required for IκBζ function [36].

In summary, IκBζ regulates pro-inflammatory cytokines released by mononuclear phagocytes in response to *S*. *pneumoniae*, with NFκB and p38 MAPK as upstream factors. Thus, the regulation of IκBζ deserves further study as a determinant of host protections from pneumococcal pneumonia.

## Supporting Information

S1 Fig IκBζ kinetics in monocytes in response to D39. Human monocytes (10^6^ cells/ml) were infected with **(A)** D39 at an MOI of 0.1 for different time periods through 24 h or with **(B)** different MOIs of D39 for 3 h. Cell extracts were analyzed for mRNA expression using qPCR. The graphs represent the mean ± SEM of 3 independent experiments. NT stands for not treated.
(PDF)

S2 Fig Gene screening following IκBζ silencing. Human monocytes (10^6^ cells/ml) were nucleofected with scrambled siRNA control or siIκBζ followed by infection with D39 at MOI 0.1 for 3 and 6 h. **(A)** Cell extracts analyzed using immunoblotting for IκBζ and β-actin expression. PamCSK4 (5ng/ml) was used as positive control for IκBζ induction and siRNA mediated knockdown. **(B)** IL-8 mRNA and protein expression in the cells, analyzed using qPCR and ELISA respectively. **(C)** qPCR for mRNA expression of various pneumonia relevant, host defense genes. The immunoblot represents 3 independent experiments and the bar graphs represent the mean ± SEM of 3 independent experiments. NT stands for not treated.
(PDF)

S3 Fig Schematic of the possible mechanism of IκBζ mediated inflammation in response to *S*.*pneumoniae*. Wildtype *S*.*pneumoniae* is sensed by the TLR1/2 receptor complex on the cell membrane of monocytes, to activate p38MAPK and NFκB, both of which are required for the primary immune response involving IκBζ. This IκBζ then activates the transcription of secondary response cytokines IL-6 and GMCSF. A TLR1/2 agonist such as LTA may be the pneumococcal pathogenic factor responsible for this immune response. The transcription factors NFκB and AP-1 could bind to IκBζ to induce the expression of IL-6 and GMCSF in response to pneumococcus.
(PDF)

S4 Fig Monocytic cell death due to pneumococcal overgrowth. Percentage LDH released from monocytes infected with D39 for different time points, as an indicator of cell death. TritonX treated cells were used as positive controls for 100% LDH release. LDH release by non-treated control was subtracted out from all the time points. The bar graph represents the mean ± SEM of 3 independent experiments. NT stands for not treated.
(PDF)

S1 Table Primer sequences for genes analyzed using quantitative real-time PCR. (PPTX)
