# Supplementary figures and images for: IκBζ Regulates Human Monocyte Pro-Inflammatory Responses Induced by Streptococcus pneumoniae
Source: PLoS One. 2016 Sep 6;11(9):e0161931. doi: 10.1371/journal.pone.0161931 (PMC5012667; doi:10.1371/journal.pone.0161931)

**A.**

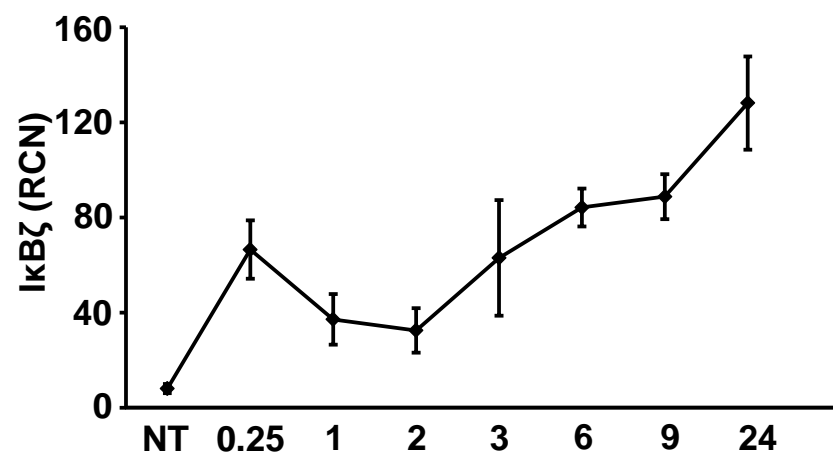

**B.**

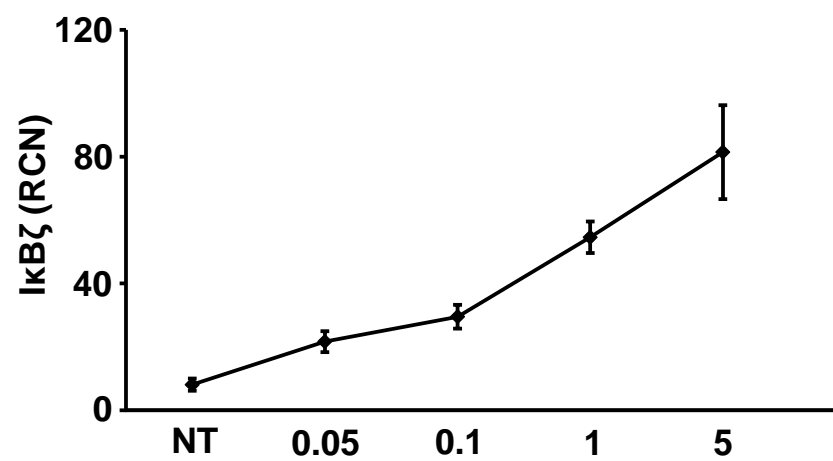

**Sup Fig S1**

Supplement: S1 Fig — Human monocytes (106 cells/ml) were infected with (A) D39 at an MOI of 0.1 for different time periods through 24 h or with (B) different MOIs of D39 for 3 h. Cell extracts were analyzed for mRNA expression using qPCR. The graphs represent the mean ± SEM of 3 independent experiments. NT stands for not treated. (PDF) [file pone.0161931.s001.pdf]

**A.**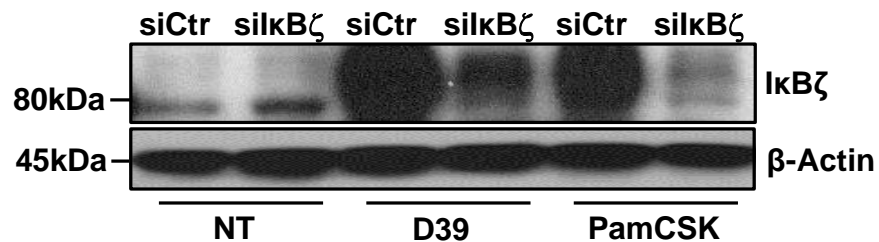**B.**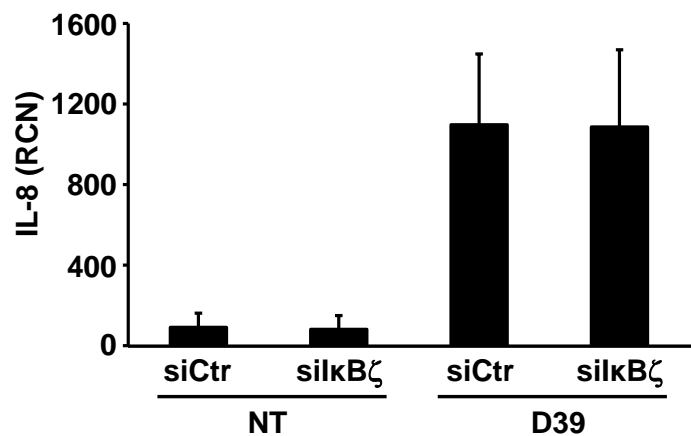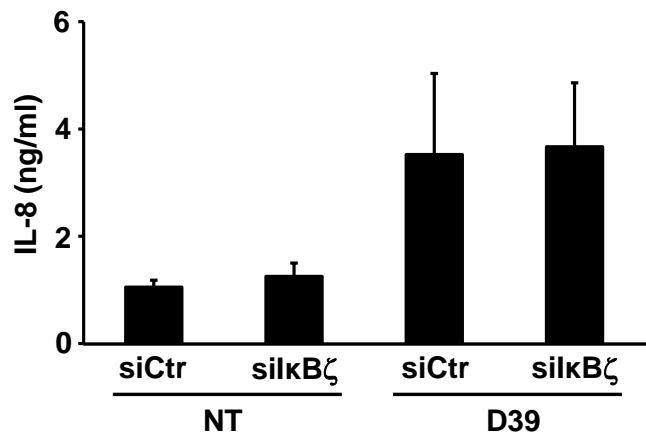**C.**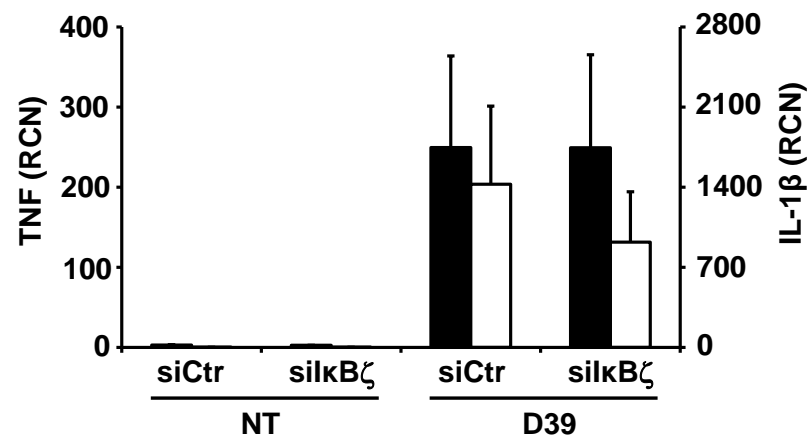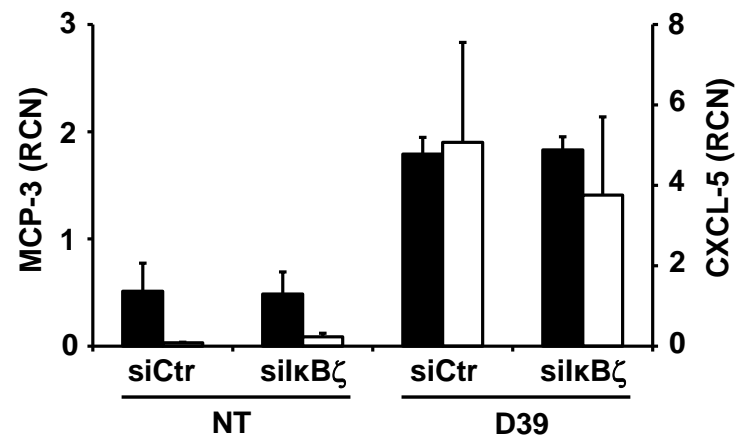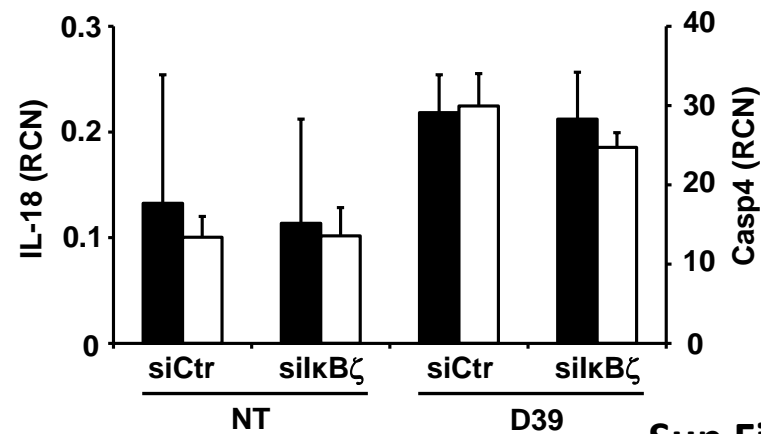

Supplement: S2 Fig — Human monocytes (106 cells/ml) were nucleofected with scrambled siRNA control or siIκBζ followed by infection with D39 at MOI 0.1 for 3 and 6 h. (A) Cell extracts analyzed using immunoblotting for IκBζ and β-actin expression. PamCSK4 (5ng/ml) was used as positive control for IκBζ induction and siRNA mediated knockdown. (B) IL-8 mRNA and protein expression in the cells, analyzed using qPCR and ELISA respectively. (C) qPCR for mRNA expression of various pneumonia relevant, host defense genes. The immunoblot represents 3 independent experiments and the bar graphs represent the mean ± SEM of 3 independent experiments. NT stands for not treated. (PDF) [file pone.0161931.s002.pdf]

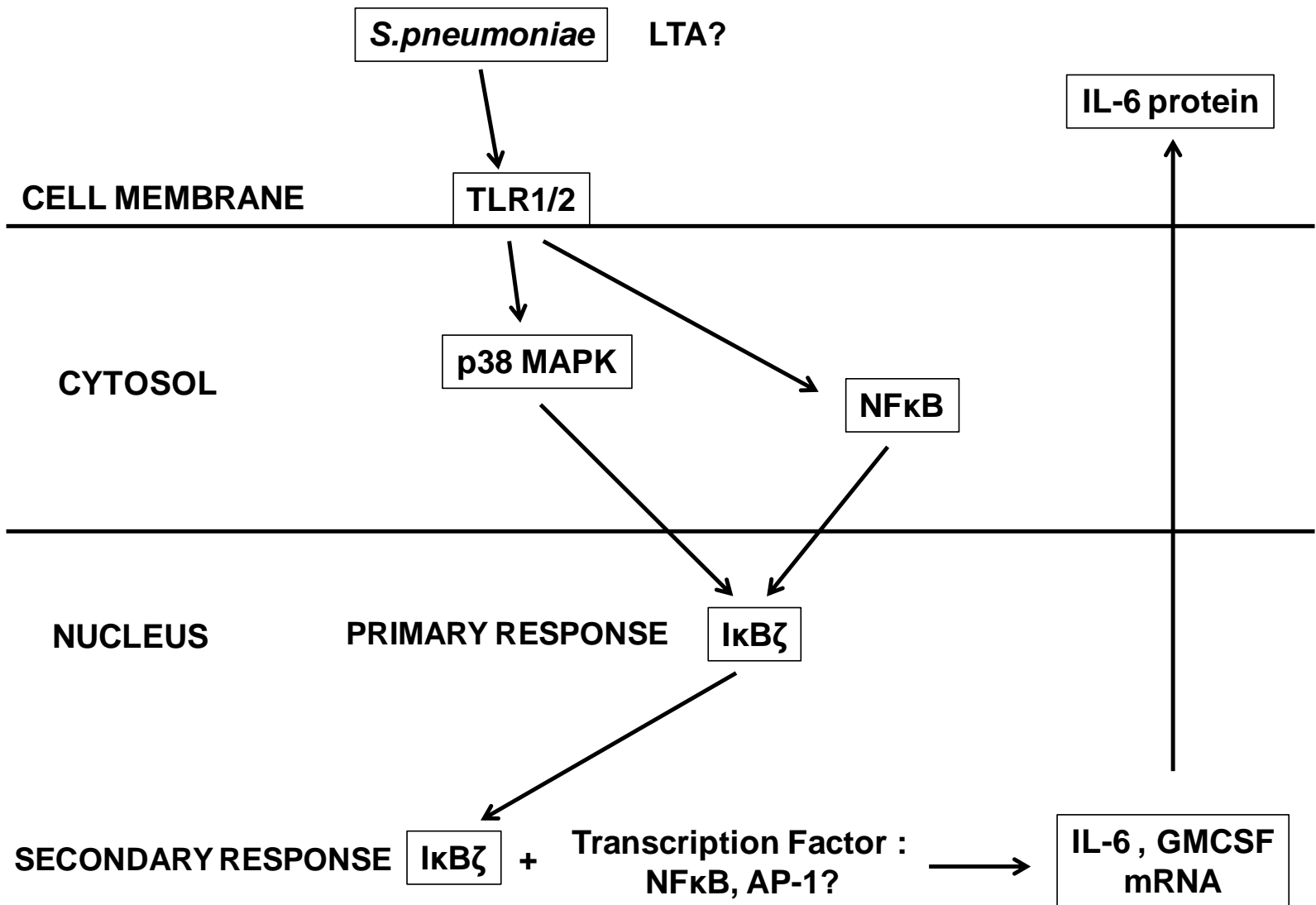

Sup Fig S3

Supplement: S3 Fig — Wildtype S.pneumoniae is sensed by the TLR1/2 receptor complex on the cell membrane of monocytes, to activate p38MAPK and NFκB, both of which are required for the primary immune response involving IκBζ. This IκBζ then activates the transcription of secondary response cytokines IL-6 and GMCSF. A TLR1/2 agonist such as LTA may be the pneumococcal pathogenic factor responsible for this immune response. The transcription factors NFκB and AP-1 could bind to IκBζ to induce the expression of IL-6 and GMCSF in response to pneumococcus. (PDF) [file pone.0161931.s003.pdf]

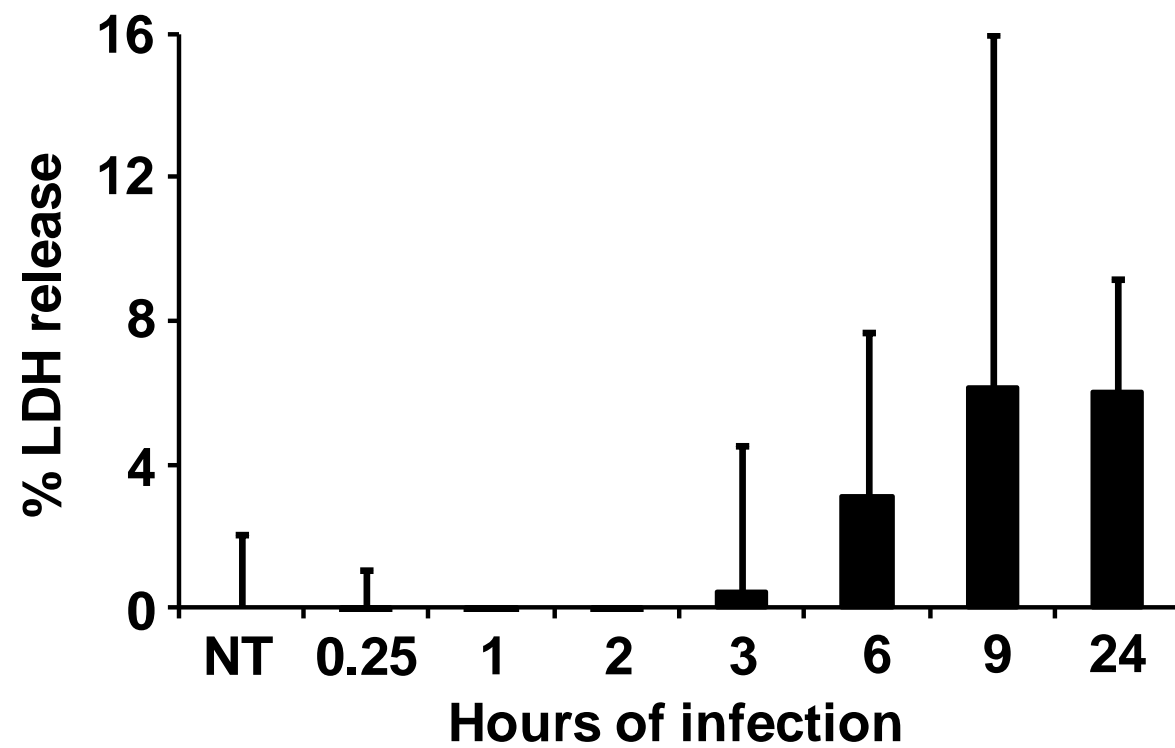

Supplement: S4 Fig — Percentage LDH released from monocytes infected with D39 for different time points, as an indicator of cell death. TritonX treated cells were used as positive controls for 100% LDH release. LDH release by non-treated control was subtracted out from all the time points. The bar graph represents the mean ± SEM of 3 independent experiments. NT stands for not treated. (PDF) [file pone.0161931.s004.pdf]
