# Supplementary material for: IκBζ Regulates Human Monocyte Pro-Inflammatory Responses Induced by Streptococcus pneumoniae
Source: PLoS One. 2016 Sep 6;11(9):e0161931. doi: 10.1371/journal.pone.0161931 (PMC5012667; doi:10.1371/journal.pone.0161931)
Supplement: S1 Table — (PPTX) [file pone.0161931.s005.pptx]

## Slide 1
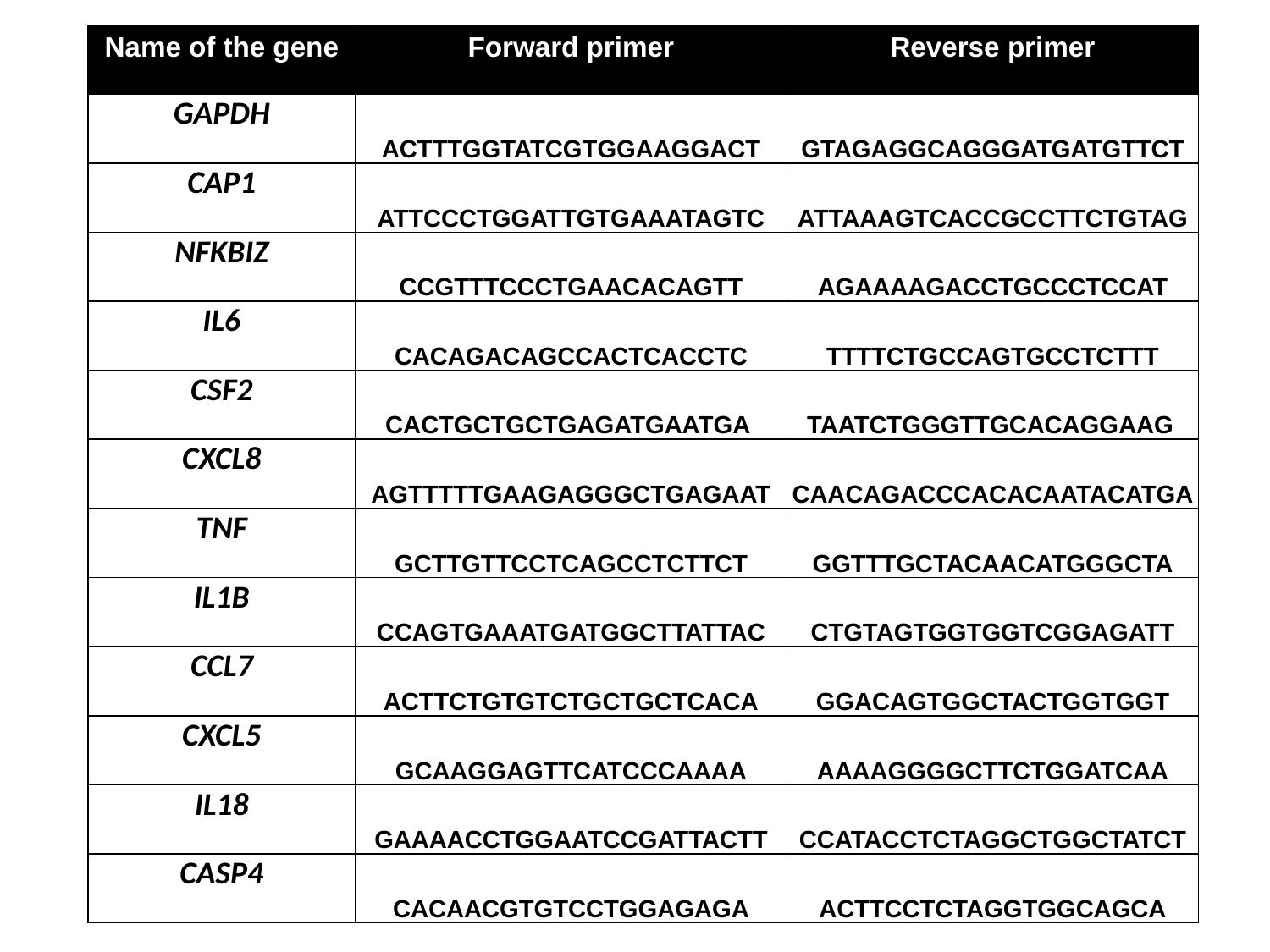

| Name of the gene | Forward primer | Reverse primer |
| --- | --- | --- |
| GAPDH | ACTTTGGTATCGTGGAAGGACT | GTAGAGGCAGGGATGATGTTCT |
| CAP1 | ATTCCCTGGATTGTGAAATAGTC | ATTAAAGTCACCGCCTTCTGTAG |
| NFKBIZ | CCGTTTCCCTGAACACAGTT | AGAAAAGACCTGCCCTCCAT |
| IL6 | CACAGACAGCCACTCACCTC | TTTTCTGCCAGTGCCTCTTT |
| CSF2 | CACTGCTGCTGAGATGAATGA | TAATCTGGGTTGCACAGGAAG |
| CXCL8 | AGTTTTTGAAGAGGGCTGAGAAT | CAACAGACCCACACAATACATGA |
| TNF | GCTTGTTCCTCAGCCTCTTCT | GGTTTGCTACAACATGGGCTA |
| IL1B | CCAGTGAAATGATGGCTTATTAC | CTGTAGTGGTGGTCGGAGATT |
| CCL7 | ACTTCTGTGTCTGCTGCTCACA | GGACAGTGGCTACTGGTGGT |
| CXCL5 | GCAAGGAGTTCATCCCAAAA | AAAAGGGGCTTCTGGATCAA |
| IL18 | GAAAACCTGGAATCCGATTACTT | CCATACCTCTAGGCTGGCTATCT |
| CASP4 | CACAACGTGTCCTGGAGAGA | ACTTCCTCTAGGTGGCAGCA |
